# Flexible energy-saving strategies in female temperate-zone bats

**DOI:** 10.1007/s00360-022-01452-7

**Published:** 2022-08-08

**Authors:** Lara Keicher, J. Ryan Shipley, Ewa Komar, Ireneusz Ruczyński, Paul J. Schaeffer, Dina K. N. Dechmann

**Affiliations:** 1 Max Planck Institute of Animal Behavior, Am Obstberg 1, 78315 Radolfzell, Germany; 2 Department of Biology, University of Konstanz, Universitätsstraße 10, 78457 Constance, Germany; 3 Mammal Research Institute, Polish Academy of Sciences, 17-230 Białowieża, Poland; 4 Department of Biology, Miami University, 700 E. High St., Oxford, OH 45056 USA

**Keywords:** Metabolism, *Nyctalus noctula*, Reproduction, Heart rate, Thermoregulation, Torpor

## Abstract

Torpor is characterized by an extreme reduction in metabolism and a common energy-saving strategy of heterothermic animals. Torpor is often associated with cold temperatures, but in the last decades, more diverse and flexible forms of torpor have been described. For example, tropical bat species maintain a low metabolism and heart rate at high ambient and body temperatures. We investigated whether bats (*Nyctalus noctula*) from the cooler temperate European regions also show this form of torpor with metabolic inhibition at high body temperatures, and whether this would be as pronounced in reproductive as in non-reproductive bats. We simultaneously measured metabolic rate, heart rate, and skin temperature in non-reproductive and pregnant females at a range of ambient temperatures. We found that they can decouple metabolic rate and heart rate from body temperature: they maintained an extremely low metabolism and heart rate when exposed to ambient temperatures changing from 0 to 32.5 °C, irrespective of reproductive status. When we simulated natural temperature conditions, all non-reproductive bats used torpor throughout the experiment. Pregnant bats used variable strategies from torpor, to maintaining normothermy, or a combination of both. Even a short torpor bout during the day saved up to 33% of the bats' total energy expenditure. Especially at higher temperatures, heart rate was a much better predictor of metabolic rate than skin temperature. We suggest that the capability to flexibly save energy across a range of ambient temperatures within and between reproductive states may be an important ability of these bats and possibly other temperate-zone heterotherms.

**Supplementary Information:**

The online version contains supplementary material available at 10.1007/s00360-022-01452-7.

## Introduction

Most organisms are energetically limited by resource availability. This has led to behavioral and physiological adaptations that involve using resources efficiently, optimizing metabolic processes, and minimizing energy expenditure. One of the most efficient strategies to minimize energy expenditure in heterothermic endotherms is torpor. Torpor is a controlled reduction of metabolic rate (Stawski et al. 2014) associated with a reduction in heart rate (Ruf and Geiser 2015; Geiser 2021). Most often, this reduced metabolism is associated with reduced body temperature when animals thermoconform to low ambient temperatures (Geiser 2021).

Torpor is commonly used by small heterothermic mammals from the temperate-zones such as ground squirrels, dormice, or bats (Geiser 2004). Selection for efficient energy-saving strategies is thought to be especially strong on temperate-zone bats due to the high costs of active flight (Schmidt-Nielsen 1970), and also because many species do not accumulate much body fat except in the pre-hibernation season (Kunz et al. 1998). The energy intake of bats is unpredictable as they often feed on ephemeral insect swarms that fluctuate in availability both in time and space (Ruczyński et al. 2020). To compensate for this, many bats select roosts with relatively low and stable ambient temperatures such as caves and tree cavities (Kunz 1982). This is thought to facilitate torpor enabling bats to reduce their body temperature and metabolism more than would be possible at higher ambient temperatures (Hamilton and Barclay 1994; Geiser 2004).

In contrast, tropical or desert bat species often experience high ambient day temperatures. This limits how far they can lower body temperature when using torpor during the day, but significant energetic savings at temperatures above the thermoneutral zone (TNZ) have also been found (Maloney et al. 1999; Bondarenco et al. 2014; Reher and Dausmann 2021). For example, the metabolism of Commerson's leaf-nosed bats (*Macronycteris commersoni*) is 80% lower at ambient temperatures of more than 41 °C compared to their resting metabolic rate (RMR; Reher and Dausmann 2021). In Pallas’ free-tailed bats (*Molossus molossus)* similar energy savings of 60% are associated with extremely low heart rates at high ambient and thus body temperatures (Dechmann et al. 2011; O'Mara et al. 2017a). These studies were only able to detect this state of low energy consumption at high ambient temperatures using direct measurements of metabolic rate or high-resolution data on heart rate (O'Mara et al. 2017a; Reher and Dausmann 2021), instead of pre-defined temperature thresholds (Audet and Fenton 1988; Barclay et al. 1996; Jonasson and Willis 2012).

Ambient temperatures can influence the depth and frequency of torpor bouts, but additional constraints on torpor use likely exist for both sexes during reproduction. In the temperate zone, sperm-producing males as well as pregnant and lactating females use torpor less frequently and in shorter and shallower bouts than non-reproductive individuals (Grinevitch et al. 1995; Lausen and Barclay 2003; Dzal and Brigham 2013; Komar et al. 2020). This is likely because extended torpor inhibits sperm development, embryonic growth, and milk production (Hamilton and Barclay 1994; Dietz and Kalko 2006; Adams 2010).

We aimed to determine if bats from temperate regions can also maintain a low metabolism and heart rate at high ambient temperatures to save energy during the reproductive and non-reproductive season. We then wanted to quantify how variation in torpor use translates into energy expenditure in reproductive and non-reproductive bats. We worked with reproductive and non-reproductive female common noctule bats (*Nyctalus noctula*). *Nyctalus noctula* is a 30 g insectivorous European bat that regularly uses torpor (Braun and Dieterlen 2003). Their short activity period with on average 2 h of foraging and the ephemerality of their insect prey likely increases the pressure to conserve energy, especially during energetically challenging reproduction. We exposed bats to varying ambient temperatures while simultaneously monitoring three physiological parameters: metabolic rate, heart rate, and skin temperature. We tested two hypotheses: (1) regardless of reproductive status, *N. noctula* are able to use torpor, reducing metabolism and heart rates at high ambient and thus body temperatures; and (2) under natural temperature conditions, reproductive individuals enter torpor less frequently and maintain higher metabolic and heart rates than non-reproductive individuals to enable fetal development. In addition, we quantified how variation in torpor use translates into energy expenditures in reproductive and non-reproductive *N. noctula* and re-examined previous results showing that heart rate is a better predictor of oxygen consumption than skin temperature.

## Materials and methods

### Study populations and bat capture

Female *N. noctula* migrate and are not present in the hibernation area during the reproductive season in summer (Dechmann et al. 2014, 2017; Lehnert et al. 2018). Thus, we conducted our study with individuals from two different populations. We worked with non-reproductive female *N. noctula* from the population in Southern Germany (hereafter “Konstanz”, 47°39′59.8′′, N 9°10′53.6′′ E) just after hibernation (post-hibernation; April 3rd–16th 2019; *n* = 10; mean body mass = 27.22 g) and just before hibernation (pre-hibernation; October 1st–17th 2019; *n* = 9; mean body mass = 33.14 g). We studied reproductive female bats from the population in the Białowieża Primeval Forest in Eastern Poland (hereafter “Białowieża”, 52°41′59.9′′ N, 23°52′04.5′′ E) during early pregnancy (May 18th–31st 2019; n = 12; mean body mass = 32.42 g). In Konstanz, we removed the bats from bat boxes during the day and transported them in individual soft cloth bags to a laboratory facility at the nearby Max Planck Institute of Animal Behavior. In Białowieża, we caught females emerging from a maternity colony at dusk with mist nets (Ecotone, Gdynia, Poland) and transported them to the nearby Mammal Research Institute of the Polish Academy of Sciences. All bats were adult at the time of capture.

After transport to the laboratory, we weighed bats with a digital scale (± 0.01 g; Kern & Sohn, Bahlingen, Germany). With each bat, we performed two respirometry experiments with different temperature regimes (see “Experimental design”). When not involved in an experiment, we kept single individuals in artificial roosts in hollow tree trunks (Ruczyński et al. 2007). Bats received mealworms and water ad libitum during their natural foraging time at dusk and we weighed them every day before and after each experiment. Bats were kept for a maximum of three days after which we released them into the box they had been removed from in Konstanz or at the capture site in Białowieża.

### Experimental design

Bats were placed into the respirometry chambers between 21:00 and 23:30 the night prior to each experiment to acclimate. We then began experiments at 06:00 the following morning. In the first respirometry experiment (“6 h-experiment”), we tested our first hypothesis that regardless of reproductive status, *N. noctula* are able to use torpor, reducing metabolism and heart rates (*f*_H_) in spite of high ambient (*T*_a_) and thus body temperatures (*T*_skin_). We exposed bats to a range of temperatures in six increasing increments of *T*_a_ (0, 7.5, 15, 22.5, 27.5, 32.5 °C) for 1 h each (06:00–12:00). *T*_a_ was measured using iButtons (DS1922L, Maxim Integrated Products, San Jose, California, USA) inside the small plastic containers used for the respirometry experiments (see below). To address our second hypothesis, that under simulated natural temperature conditions reproductive individuals would use torpor less frequently and maintain higher metabolic rates than non-reproductive individuals, we conducted a second experiment (“12 h-experiment”). We averaged *T*_a_ from Białowieża and Konstanz from the previous 2 years for each month and calculated means for 4-h time periods during the day and a single mean temperature during the night (06:00–10:00, 10:00–14:00, 14:00–18:00, and 18:00–06:00). Resulting experimental *T*_a_ during non-reproduction were 9.1 °C, 13.6 °C, 15.0 °C and 9.0 °C (post-hibernation), and 9.1 °C, 12.2 °C, 12.8 °C and 9.2 °C (pre-hibernation) and during reproduction 13.4 °C, 17.5 °C, 18.6 °C and 12.7 °C (pregnancy).

### Heart rate transmitter attachment and monitoring

We attached external heart rate transmitters (ca. 0.8 g, 5 × 3 × 8 mm; SP2000 HR Sparrow Systems, Fisher, Illinois, USA) to each bat (Dechmann et al. 2011; O'Mara et al. 2014, 2017a, b) a minimum of 6 h before the start of the first respirometry experiment. Each transmitter emits a continuous long-wave carrier signal which is interrupted by cardiac muscle potentials. We used receivers (AR8000, AOR Ltd, Tokyo, Japan) connected to digital recorders (Tascam DR-05, Los Angeles, California, USA) and recorded sound files of the time series of the cardiac muscle potentials continuously throughout experiments. Before attachment, transmitters were mounted on fabric with two wires extending through the fabric. We cut the dorsal fur at both wire insertion points, one between the shoulder blades and one in the left lumbar region. We disinfected the skin with 70% EtOH, then punctured it with a 23GA sterile needle and inserted the transmitter’s two disinfected wires ca. 5 mm under the skin (see illustration in O’Mara et al. 2017b). We glued both wires in place with surgical cement (Perma-Type Company, Plainville, Connecticut, USA) and then glued the fabric with the mounted transmitter to the back, covering the wire insertion points.

We used a custom R script to automatically identify the interruptions of the carrier signal by the muscle potentials and calculated *f*_H_ in beats per minute (bpm) (O’Mara et al. 2017a; b). Automatically analyzed files were visually subsampled frequently to validate the filtering method, particularly when variation in *f*_H_ was high. One observer (LK) manually counted heartbeats when automated analysis was not possible due to interference or noise.

### Skin temperature monitoring

We measured *T*_skin_ with iButtons (ca. 1.6 g, DS1922L, Maxim Integrated Products, San Jose, California, USA) modified following Lovegrove (2009). The iButtons recorded *T*_skin_ every 2 min. We glued them with surgical cement near the place of insertion of the lower wire where fur was already removed. We then covered heart rate transmitters and iButtons with a second piece of fabric to prevent the animals from scratching. After completing experiments, we removed transmitters and iButtons immediately. Bats maintained body mass while wearing the equipment, suggesting no measurable negative impact of the experiments.

### Respirometry and calculation of oxygen consumption

To measure metabolic rates, we used an open-flow pull through respirometry system with additional humidity control (Sable Systems International, Las Vegas, NV, USA). This setup allows for simultaneous analysis of O_2_, CO_2_ and water vapor pressure (WVP) from up to three individuals plus one empty control chamber for collecting baseline values. The setup was zeroed and spanned before each sampling season using laboratory reference gases. Bats were placed in small airtight plastic containers (volume = 800 mL) which contained a plastic grid wrapped in mesh. This allowed the bats to roost in a natural hanging position while allowing air circulation. Four mass flow systems (MFS, Sable Systems International, Las Vegas, NV, USA) with mass flow meters pulled humidity-controlled air (DG-4, Sable Systems International, Las Vegas, NV, USA) through a copper spiral for faster temperature equilibration with a constant flow rate of 150 mL/min through each of the four chambers. A subsampler (RM-8, Sable Systems International, Las Vegas, NV, USA) switched between chambers and O_2_, CO_2_ and WVP were analyzed with a field metabolic system (FMS, Sable Systems International, Las Vegas, NV, USA). We placed the respirometry chambers and the copper spiral into a climate-controlled incubator (KB53, BINDER GmbH, Tuttlingen, Germany) with a small opening on the lid to mimic the roost or bat box entrance and set the light regime in the room to the natural local circadian rhythm.

We calculated rates of oxygen consumption ($\dot{V}{\text{O}}_{2}$) and of CO_2_ production ($\dot{V}{\text{CO}}_{2}$) for each individual using Eqs. 11.7 and 11.8 from Lighton (2018). Before calculation of $\dot{V}{\text{O}}_{2}$ and $\dot{V}{\text{CO}}_{2}$, we removed the first 30 s after a channel switch and corrected for drift using a spline fit (Forsythe et al. 1977). The raw O_2_ and CO_2_ data were phase-corrected to account for the tubing connecting each of the different components. We calculated incurrent and excurrent fractional gas concentrations while correcting for water vapor dilution using Eq. 8.6 from Lighton (2018). We standardized V̇O_2_ based on individual body mass. We used the mean of body mass before and after the experiments in the equation to calculate $\dot{V}{\text{O}}_{2}$ and report all $\dot{V}{\text{O}}_{2}$ measurements in mL O_2_ g^−1^ h^−1^.

### Data analysis and hypothesis testing

We collected data at different temporal resolutions. The $\dot{V}{\text{O}}_{2}$ data had the lowest resolution because we switched between the respirometry chambers to measure three bats in the same experiment. This resulted in 3-min $\dot{V}{\text{O}}_{2}$ intervals every 9 min for each bat. In each 3-min interval, we calculated the mean per minute of $\dot{V}{\text{O}}_{2}$, *f*_H_, *T*_skin_, and *T*_a_. This allowed accurate conclusions about the interplay of the three different physiological parameters at changing *T*_a_. We pooled the pre- and post-hibernation data of non-reproductive bats, as we observed no difference in the physiological states bats entered in both periods and both experiments.

We defined different physiological states by visually inspecting $\dot{V}{\text{O}}_{2}$ over time for each individual in the 6-h and 12-h experiment. Because of the high variability in $\dot{V}{\text{O}}_{2}$ across different *T*_a_, we decided to use this method instead of approaches which use *T*_skin_ or $\dot{V}{\text{O}}_{2}$ thresholds. We differentiated between torpid, resting, arousal, and torpor entry states and excluded arousal and torpor entry states for further analysis. In our experiments, at *T*_a_ < 20 °C, the torpid metabolic rate of *N. noctula* was 92–96% lower than metabolic rate during resting and for *T*_a_ > 20 °C 45–85% lower than metabolic rate during resting. As both ranges lie in the normal range of metabolic depression (compared to resting metabolic rate (RMR) or basal metabolic rate (BMR)) in *N. noctula* and other heterothermic bats (84–99% at cold temperatures (Geiser 2004), 15–93% at warm temperatures (Hosken and Withers 1997; Geiser 2004; O'Mara et al. 2017a; Reher and Dausmann 2021), we are confident that we correctly assigned physiological states.

We tested the first hypothesis in the 6-h experiment, by measuring $\dot{V}{\text{O}}_{2}$, *f*_H_, and *T*_skin_ of reproductive and non-reproductive torpid bats when exposed to rising *T*_a_. Some bats showed occasional arousals which were excluded from analysis as we were focused only on the torpid states. We fitted generalized linear mixed-effect models (glmer, Gamma family, log link, package *lme4* (Bates et al. 2015)) to explore variation in response variables ($\dot{V}{\text{O}}_{2}$, *f*_H_ and *T*_skin_) based on predictor variables (*T*_a_, reproductive status) with individual (BatID) as a random factor.

We tested the second hypothesis in the 12-h experiment, by assessing the relative time reproductive and non-reproductive bats spent either in a torpid or resting state and calculating the mean energy expenditure for each bat using $\dot{V}{\text{O}}_{2}$. We fitted generalized linear mixed-effect models (Gamma family, log link) to investigate variation in response variables ($\dot{V}{\text{O}}_{2}$, *f*_H_, and *T*_skin_) based on predictor variables (reproductive status and torpid or resting state) with individual as a random factor (BatID). We calculated mean ± SD for $\dot{V}{\text{O}}_{2}$, *f*_H_ and *T*_skin_ from reproductive and non-reproductive resting and torpid bats. To assess the variation in $\dot{V}{\text{O}}_{2}$, *f*_H_ and *T*_skin_ in resting bats, we calculated the coefficients of variation (CV).

Lastly, we used data from both experiments to test whether *f*_H_ is a better predictor of oxygen consumption than *T*_skin_. We fitted linear mixed-effect models (lmer) with $\dot{V}{\text{O}}_{2}$ as the response variable and individual as a random factor (BatID) and tested if *f*_H_ or *T*_skin_ would result in a better model fit. We log-transformed $\dot{V}{\text{O}}_{2}$ to confirm equal variance and normal distribution of standardized residuals. We calculated the Akaike information criterion corrected for small sample sizes (AICc) for each model and the marginal R^2^ (R^2^m, fixed effects alone) and conditional (R^2^c, full model) values (Nakagawa and Schielzeth 2013) in *MuMIn* (Bartoń 2020). We then calculated $\dot{V}{\text{O}}_{2}$ predictions based on *f*_H_ and *T*_skin_ and compared those to measured $\dot{V}{\text{O}}_{2}$ from the 6-h experiment, to test the model performances over a wide range of *T*_a_.

All analyses were performed in R [Version R 4.1.3 (R Core Team 2022), RStudio Version 2022.02.1 (RStudio Team 2022)].

## Results

### *Nyctalus noctula* can maintain a reduced metabolism and heart rate at high ambient temperatures regardless of reproductive status (Hypothesis 1)

**Fig. 1:**
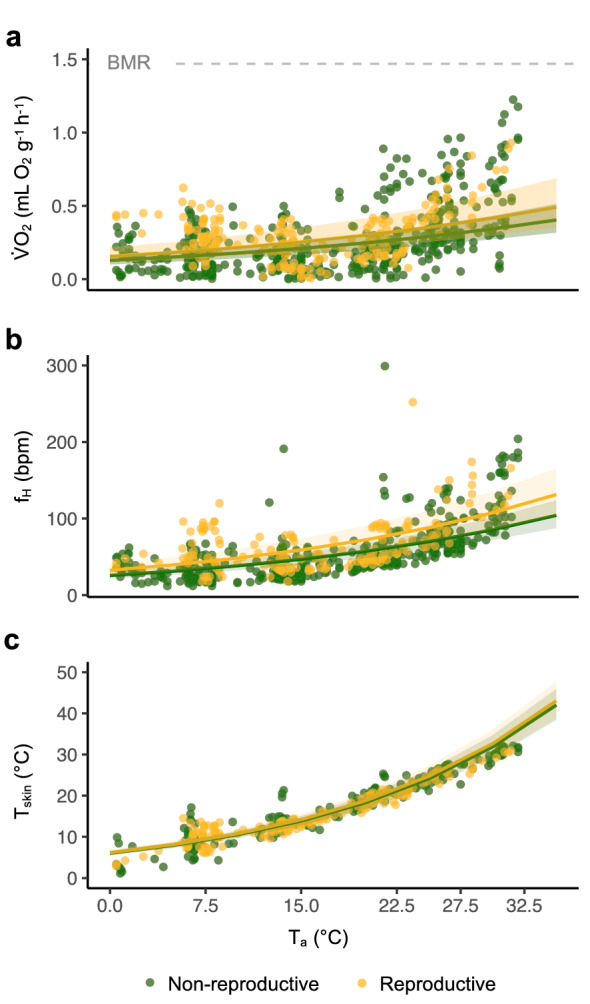
Irrespective of reproductive status (green = non-reproductive, yellow = reproductive), *N. noctula* remained torpid when exposed to rising *T*_a_. **a** $\dot{V}{\text{O}}_{2}$ increased slightly but remained low across all *T*_a_ (non-reproductive: *n* = 19, *n*_Observations_ = 491; reproductive: *n* = 9, *n*_Observations_ = 193). Grey dashed line indicates the BMR of *N. noctula* = 1.47 mL O_2_ g^−1^ h^−1^ (reported in Geiser 2004). **b** f_H_ increased slightly but remained low across a wide range of *T*_a_ (non-reproductive: *n* = 19, *n*_Observations_ = 484; reproductive: *n* = 9, *n*_Observations_ = 164). **c** *T*_skin_ increased with rising T_a_ when torpid bats thermoconformed (non-reproductive: *n* = 12, *n*_Observations_ = 256; reproductive: *n* = 9, *n*_Observations_ = 182). Shaded green and yellow areas indicate the 95% CI

When T_a_ was raised in hourly increments from 0 to 32.5 °C in the 6-h experiment, we found overall low $\dot{V}{\text{O}}_{2}$ and *f*_H_ (Figs. 1a, b, S1a) in torpid reproductive and non-reproductive bats. In reproductive bats, a mean $\dot{V}{\text{O}}_{2}$ of 0.29 ± 0.17 mL O_2_ g^−1^ h^−1^ (*n* = 9, *n*_Observations_ = 193) and, in non-reproductive bats, we found a mean $\dot{V}{\text{O}}_{2}$ of 0.26 ± 0.22 mL O_2_ g^−1^ h^−1^ (*n* = 19, *n*_Observations_ = 491). The predicted relationship between $\dot{V}{\text{O}}_{2}$ and *T*_a_ for reproductive bats was $\dot{V}{\text{O}}_{2}$ = exp(− 1.86 + 0.03 × *T*_a_) and for non-reproductive bats $\dot{V}{\text{O}}_{2}$ = exp(− 2.05 + 0.03 × *T*_a_). In reproductive bats, a mean *f*_H_ of 64 ± 34 bpm (*n* = 9, *n*_Observations_ = 164) and, in non-reproductive bats, we found a mean *f*_H_ of 55 ± 36 bpm (*n* = 19, *n*_Observations_ = 484). The predicted relationship between *f*_H_ and T_a_ for reproductive bats was *f*_H_ = exp(3.48 + 0.04 × *T*_a_) and for non-reproductive bats *f*_H_ = exp(3.25 + 0.04 × *T*_a_). Bats thermoconformed to T_a_ (Fig. 1c) and we recorded a wide range of T_skin_ (reproductive: 2.9–30.9 °C, *n* = 9, *n*_Observations_ = 182; non-reproductive: 1.1–32.2 °C, *n* = 12, *n*_Observations_ = 256). The predicted relationship between *T*_skin_ and *T*_a_ for reproductive bats was *T*_skin_ = exp(1.82 + 0.06 × *T*_a_) and for non-reproductive bats *T*_skin_ = exp(1.80 + 0.06 × *T*_a_). There were occasional arousals (Fig. S1b) which were excluded from analysis. Overall, *T*_a_ explained the variation in response variables ($\dot{V}{\text{O}}_{2}$, *f*_H_ and *T*_skin_) while reproductive status had no significant effect (Table S1).

### Under natural temperature conditions, reproductive bats use torpor less frequently than non-reproductive bats (Hypothesis 2)

**Fig. 2:**
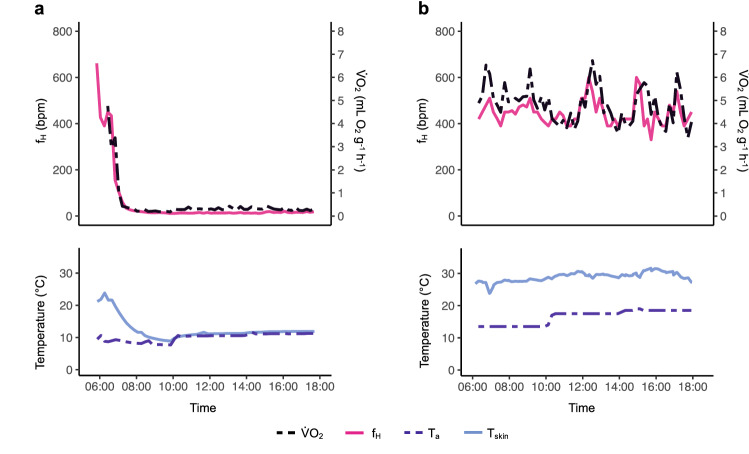
Different torpor use strategies in non-reproductive and reproductive female *N. noctula* in the 12-h experiment. **a** Representative figure of a non-reproductive bat using the “only torpid” strategy. Upper panel: $\dot{V}{\text{O}}_{2}$ (black dashed line) and *f*_H_ (pink solid line) were lowered after a short arousal at the beginning of the experiment. Lower panel: the bat thermoconformed *T*_skin_ (light-blue solid line) to *T*_a_ (dark-blue dashed line). **b** Representative figure of a reproductive bat using the “only resting” strategy. Upper panel: $\dot{V}{\text{O}}_{2}$ (black dashed line) and *f*_H_ (pink solid line) were very variable. Lower panel: the bat thermoregulated and *T*_skin_ (light-blue solid line) was constantly higher than *T*_a_ (dark-blue dashed line)

We exposed bats to seasonal *T*_a_ in the 12-h experiment to assess the physiological responses in non-reproductive and reproductive bats. After a short arousal at the beginning of the experiment, all non-reproductive bats (*n* = 19) had low *T*_skin_, *f*_H_, and $\dot{V}{\text{O}}_{2}$ and thermoconformed to *T*_a_ throughout the experiment (“Only torpid”) (Fig. 2a). In reproductive bats (*n* = 12), we found three different torpor use strategies: Four individuals rested throughout the 12-h experiment (“Only resting”, Fig. 2b) and had the highest mean $\dot{V}{\text{O}}_{2}$ ± SD, three individuals exclusively used torpor throughout the experiment (“Only torpid”) and had 93% energy savings compared to the “only resting” bats and five individuals used a combination of resting and torpor (“Combination”, Fig. S2) which resulted in lower mean $\dot{V}{\text{O}}_{2}$ ± SD and 33% energy savings (Table 1).

**Table 1:** Torpor use strategies by reproductive and non-reproductive bats (*n* = sample size) in the 12-h experiment

Reproductive status	Torpor use strategy	*n*	Mean $\dot{V}{\text{O}}_{2}$ ± SD (mL O_2_ g^−1^ h^−1^)	% Energy savings compared to reproductive “Only resting”
Reproductive	Only resting	4	4.99 ± 0.70	–
Reproductive	Combination	5	3.34 ± 1.16	33%
Reproductive	Only torpid	3	0.37 ± 0.06	93%
Non-reproductive	Only torpid	19	0.15 ± 0.11	97%

Reproductive bats used three different strategies which resulted in large differences in energetic savings

Reproductive status and whether the bats were in a torpid or resting state explained variation in $\dot{V}{\text{O}}_{2}$, *f*_H_ and *T*_skin_ with significant differences between resting reproductive, torpid reproductive and torpid non-reproductive individuals (Table S2). Mean ± SD of $\dot{V}{\text{O}}_{2}$, *f*_H_ and *T*_skin_ was highest in resting reproductive individuals. In torpid bats, mean ± SD of $\dot{V}{\text{O}}_{2}$ and *f*_H_ were higher in reproductive individuals, compared to non-reproductive individuals (Table 2). The differences in *T*_skin_ in torpid bats were explained by the different *T*_a_ the bats were exposed to in each season. Resting individuals had the highest and most variable $\dot{V}{\text{O}}_{2}$ with a coefficient of variation (CV) of 22.66 and *f*_H_ with a CV of 16.23 compared to *T*_skin_ with a CV of 7.93.

**Table 2:** Mean ± SD for $\dot{V}{\text{O}}_{2}$, *f*_H_ and *T*_skin_ of reproductive and non-reproductive bats in a resting or torpid state

Reproductive status	State	*n*	$\dot{V}{\text{O}}_{2}$ ± SD (mL O_2_ g^−1^ h^−1^)	*f*_H_ ± SD (bpm)	*T*_skin_ ± SD (°C)
Reproductive	Resting	9	5.02 ± 1.14	456 ± 74	28.2 ± 2.2
Reproductive	Torpid	8	0.42 ± 0.16	56 ± 47	17.5 ± 1.5
Non-reproductive	Torpid	19	0.16 ± 0.13	21 ± 8	11.4 ± 2.2

For all three parameters we found significant differences between reproductive resting and torpid bats, between reproductive and non-reproductive torpid bats, and between reproductive resting and non-reproductive torpid bats (Table S2)

Note that some reproductive bats were for some time in the experiment both in a torpid and resting state and, therefore, those animals contributed to the *n* in both states

### Heart rate as a predictor of oxygen consumption

**Fig. 3:**
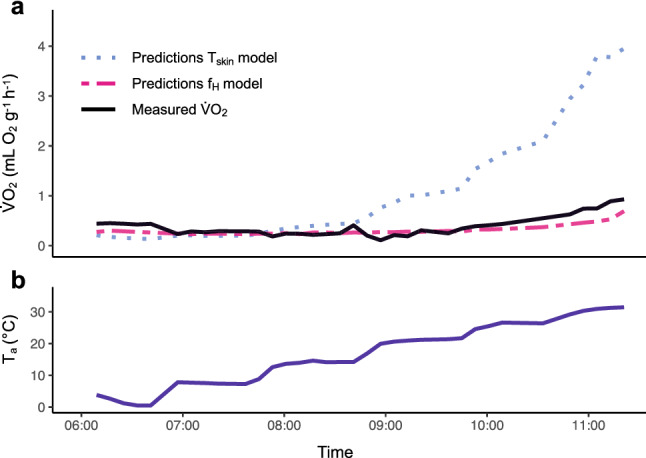
Predictions for $\dot{V}{\text{O}}_{2}$ from the *T*_skin_ model and the *f*_H_ model and the measured $\dot{V}{\text{O}}_{2}$ for one representative bat in the 6-h experiment with rising *T*_a_. **a** Predictions for $\dot{V}{\text{O}}_{2}$ from the *f*_H_ model (pink dashed line) were similar to the measured $\dot{V}{\text{O}}_{2}$ (black solid line). Predictions for $\dot{V}{\text{O}}_{2}$ from the *T*_skin_ model (light-blue dotted line) overpredicted $\dot{V}{\text{O}}_{2}$ when *T*_a_ was raised above 20 °C. **b** *T*_a_ (dark-blue solid line) was raised every hour and was > 20 °C after 09:00

We fitted linear mixed-effect models based on data from both experiments to verify that *f*_H_ is a better predictor of $\dot{V}{\text{O}}_{2}$ than *T*_skin_. The model that included *f*_H_ (*f*_H_ model) had a high R^2^c (0.83) and lower AICc and explained the variation in $\dot{V}{\text{O}}_{2}$ well. The model that included *T*_skin_ (*T*_skin_ model) had a lower predictive ability for $\dot{V}{\text{O}}_{2}$ (Table S3). We then predicted values from the *T*_skin_ model (traditionally used method) and the *f*_H_ model and compared them to measured $\dot{V}{\text{O}}_{2}$ values from bats in the 6-h experiment. When T_a_ > 20 °C, the *T*_skin_ model overpredicted $\dot{V}{\text{O}}_{2}$ while the *f*_H_ model better predicted $\dot{V}{\text{O}}_{2}$ (Fig. 3).

## Discussion

Consistent with our hypotheses, we found frequent use of torpor at high *T*_a_ with low $\dot{V}{\text{O}}_{2}$ and f_H_ but high *T*_skin_, in our temperate-zone bats. We demonstrated the wide-ranging energetic consequences of the variability in torpor use on reproductive animals and confirmed that *f*_H_ is a better predictor for $\dot{V}{\text{O}}_{2}$ than *T*_skin_.

At high *T*_a_, female *N. noctula* reduced $\dot{V}{\text{O}}_{2}$ by up to 85% compared to BMR (reported in Geiser 2004) (Fig. 1a) while *f*_H_ was reduced to 1/8 of resting *f*_H_ (Fig. 1b). Although *T*_skin_ remained high (Fig. 1c), this physiological state would be defined as torpor (Geiser 2021). A similar torpid state at high *T*_a_ is known from tropical bat species (*M. molossus*: 60% decrease in metabolic rate and 90% in *f*_H_ (O'Mara et al. 2017a) or *M. commersoni* [80% decrease in metabolic rate, *f*_H_ not measured (Reher and Dausmann 2021)], but has never been described in a temperate-zone bat. Heterothermy most likely evolved in the tropics and is considered to be an evolutionary stage between the ancestral ectothermy and the endothermy found in most mammal species (Grigg et al. 2004; Lovegrove 2017). It is, therefore, possible that temperate-zone bat species also preserved the physiological ability to express torpor at higher *T*_a_, despite usually experiencing cooler *T*_a_ than their tropical counterparts.

The physiological mechanisms which allow *N. noctula* to maintain a reduced $\dot{V}{\text{O}}_{2}$ and *f*_H_ at high *T*_skin_ are unknown. In our experiment, bats entered torpor at cold *T*_a_, but *T*_skin_ was decoupled from *f*_H_ and $\dot{V}{\text{O}}_{2}$ with rising *T*_a_. We observed a slight increase in *f*_H_ and $\dot{V}{\text{O}}_{2}$ with rising *T*_a_; however, $\dot{V}{\text{O}}_{2}$ at 32.5 °C was still far below the BMR (Figs. 1a, S1). This suggests that the reduced metabolism observed during torpor was not just a consequence of passive thermal effects (Guppy and Withers 1999), where one would expect that increases in *T*_a_ and *T*_skin_ translate directly to increased $\dot{V}{\text{O}}_{2}$. Instead, the reduced metabolism was likely a consequence of an active metabolic depression (Heldmaier et al. 2004; Geiser 2021). It remains unknown if tropical and temperate-zone bat species use the same underlying physiological processes for this. Additionally, while cardiac function during torpor at low body temperatures has been investigated (Milsom et al. 2001; Currie et al. 2018), the dynamics of blood pressure, blood viscosity, stroke volume and heart muscle function when the heart performs at different paces and extreme differences in body temperatures have not been evaluated.

We exposed captive bats to rising *T*_a_ with an increase of 32.5 °C within 6 h; a scenario which is unlikely to occur naturally. Nevertheless, the fact that reproductive and non-reproductive individuals had the physiological ability to decouple f_H_ and $\dot{V}{\text{O}}_{2}$ from *T*_skin_ suggests that this strategy is also available to bats in the wild. The maximum heat tolerance in *N. noctula* is unknown, but during summer *N. noctula* seem to prefer natural tree cavities over artificial roosting boxes in Konstanz (personal observation). They possibly avoid high *T*_a_ with this behavior, as tree-cavity roosting temperate-zone bat species generally have lower heat tolerance (Noakes et al. 2021) and at the population level, high summer daytime temperatures are associated with higher mortality (Mundinger et al. 2021). However, in a population in Białowieża, Poland, pregnant females prefer warmer maternity roosts, potentially to decrease energy expenditure when remaining normothermic (Ruczyński 2006; Ruczyński and Bartoń 2020). The factors which determine when a bat uses torpor at high body temperatures are unknown, but we suggest that using torpor at higher *T*_a_ is energetically less costly and reduces water loss compared to maintaining a high metabolism. Further study with a combination of monitoring roost temperatures and *f*_H_ of free-ranging bats could help quantify how often bats are exposed to higher *T*_a_ and to what extent they use torpor as an energy-saving strategy.

When we exposed bats to simulated *T*_a_ in the 12-h experiment, all non-reproductive bats used torpor throughout (Fig. 2a), whereas reproductive bats used different torpor strategies (Table 1, Figs. 2b, S2). Notably, following outside temperatures, we did not expose the bats to *T*_a_ higher than 18.6 °C in the 12-h experiment and they did not use torpor at high *T*_a_. The fact that reproductive bats use less torpor than non-reproductive ones is well known and most likely due to negative impacts on foetal development or milk production (Hamilton and Barclay 1994; Grinevitch et al. 1995; Dietz and Kalko 2006; Rambaldini and Brigham 2008; Dietz and Hörig 2011; Baloun and Guglielmo 2019). It is more difficult to ascertain why there is individual variation in torpor use in reproductive individuals (Rambaldini and Brigham 2008; Besler and Broders 2019). In other heterothermic species, different torpor use strategies within one reproductive stage are the result of various factors such as *T*_a_ (Geiser and Broome 1993; Dausmann et al. 2020), roost type (Rintoul and Brigham 2014), foraging success or food availability (Canale et al. 2011; Vuarin and Henry 2014; Komar et al. 2020), genetic variation in torpor related traits and body condition (Lane et al. 2011; Vuarin et al. 2013), state of pregnancy (Besler and Broders 2019), age (Bieber et al. 2018), and differences in personality or stress responses under laboratory conditions (Ruf and Geiser 2015). In our experiment, all bats from the same season were fed ad libitum and exposed to identical *T*_a_ and light and yet reproductive females differed in torpor use. We did not find any common characteristics, such as body condition or age among the three reproductive females which always used torpor, which would explain why their strategy differed from the other reproductive bats. We need further research to fully understand if and how body condition, age, stress or genetic variation affects torpor use strategies in heterothermic animals.

The different torpor use strategies we observed resulted in large differences in energy expenditure. Reproductive females using torpor had slightly higher *f*_H_ and $\dot{V}{\text{O}}_{2}$ than torpid non-reproductive individuals (Table 2). This could be a consequence of differences in metabolic rates between reproductive and non-reproductive female bats (McLean and Speakman 2000). However, when kept under identical thermal conditions, torpid metabolic rate did not differ across reproductive stages in another bat species (Turbill and Geiser 2006), and it is likely that the differences in metabolic rate are a consequence of the higher *T*_a_ (matching seasonal ambient conditions) reproductive bats were exposed to in the 12-h experiment. “Only torpid” reproductive individuals saved 93% of their daily energy expenditure compared to “only resting” individuals and the bats which used the “combination” strategy saved 33% (Table 1). This shows that when reproductive individuals use torpor even for part of the day, they can save large amounts of energy while potentially keeping the detrimental effects on reproduction low. One possibility to explain the variation in our results is that females may flexibly allocate energy to foetal growth based on individual energetic levels. Indeed, *T*_skin_ monitoring in free-ranging individuals indicates that female bats flexibly choose a torpor use strategy and opportunistically use torpor to decrease energy expenditure in different reproductive states depending on environmental conditions and possibly feeding success (Lausen and Barclay 2003; Dzal and Brigham 2013).

Even though our results from captive bats seem to fit observations of torpor use in free-ranging bats, we cannot exclude the possibility that captivity affected the bats' torpor use (Geiser et al. 2000). We, therefore, want to emphasize the importance to study free-ranging individuals to reveal the full potential of the various strategies bats use to optimize energy expenditure. f_H_ has been used successfully to monitor torpor of hibernators such as ground squirrels (Milsom et al. 1999; MacCannell et al. 2018) or brown bears (Evans et al. 2016) in the wild, and in the past years, equipment has become small and light enough to monitor *f*_H_ in free-ranging bats (O'Mara et al. 2017a, b). We show that in both torpid and normothermic bats, changes in *f*_H_ are almost immediately reflected in $\dot{V}{\text{O}}_{2}$ while *T*_skin_ follows more slowly and with a delay, if at all (Figs. 2, S1, S2). In both experiments, across a wide range of *T*_skin_, $\dot{V}{\text{O}}_{2}$ was strongly correlated with *f*_H_ (Figs. 1, 2) and the *f*_H_ model predicted $\dot{V}{\text{O}}_{2}$ better than the *T*_skin_ model (Table S3). Our data show that models based on measurements of *T*_skin_ allow accurate predictions for *N. noctula* when *T*_a_ are below 20 °C, but the error increases rapidly at higher *T*_a_ (Fig. 3).

Flexibility in torpor use might be key to helping heterothermic species overcome energetic challenges during reproduction and minimize the threat of extinction in an increasingly warmer world (Geiser and Turbill 2009; Canale et al. 2011; Levesque et al. 2016). Using *f*_H_ monitoring, future research can accurately quantify how the use of torpor at high body temperatures and flexible torpor use translates in daily energy expenditures in free-ranging heterothermic species. This will bring us one step closer to reveal the full extent of behavioral and physiological adaptations animals use to survive.

## Supplementary Information

Below is the link to the electronic supplementary material.

Supplementary file1 (PDF 179 KB)

## Data Availability

Data files associated with this study are deposited in the Open Research Data Repository of the Max Planck Society “Edmond” (10.17617/3.T5LUIM).
