# Supplementary material for: Flexible energy-saving strategies in female temperate-zone bats
Source: J Comp Physiol B. 2022 Aug 8;192(6):805–14. doi: 10.1007/s00360-022-01452-7 (PMC9550788; doi:10.1007/s00360-022-01452-7)
Supplement: Supplementary file 1 — Supplementary file1 (PDF 179 KB) [file 360_2022_1452_MOESM1_ESM.pdf]

Supplementary Information

Title: Flexible energy-saving strategies in female temperate-zone bats

Journal: Journal of Comparative Physiology B

Authors: Lara Keicher, J Ryan Shipley, Ewa Komar, Ireneusz Ruczyński, Paul J Schaeffer, Dina KN Dechmann

Affiliation and e-mail address of the corresponding author: Max Planck Institute of Animal Behavior, Am Obstberg 1, 78315 Radolfzell, Germany; Department of Biology, University of Konstanz, Universitätsstraße 10, 78457 Konstanz, Germany; ORCID ID: 0000-0001-5051-5588; lkeicher@ab.mpg.de

This file contains:

- Table S1 with the output of the Generalized linear mixed-effect models for hypothesis 1
- Table S2 with the output of the Generalized linear mixed-effect models for hypothesis 2
- Table S3 with the output of the Linear mixed-effect models investigating the predictive ability of heart rate and skin temperature for metabolic rate
- Figure S1 with two representative plots of the 6 h-experiment
- Figure S2 with a representative plot of a bat in the 12 h-experiment that used the “Combination” torpor use strategy

Table S1 Generalized linear mixed-effect models with Gamma family and log link: T_a explained the variation in response variables ($\dot{V}O_2$, f_H and T_{skin}) while reproductive status (Repro. status) had no significant effect. Individual (BatID) was included as a random effect. Reported p-values are based on t-statistics and use the normal distribution function (Lüdecke 2021)

	**$\dot{V}O_2$**			**f_H**			**T_{skin}**		
**Predictors**	Estimates	CI	p	Estimates	CI	p	Estimates	CI	p
T_a	1.03	1.03 - 1.04	**<0.001**	1.04	1.04 - 1.04	**<0.001**	1.59	1.56 - 1.62	**<0.001**
Repro. status [Reproductive]	1.21	0.82 - 1.80	0.329	1.26	0.95 - 1.67	0.105	1.02	0.90 - 1.16	0.704
**Random Effect [BatID]**									
σ^2	0.33			0.15			0.03		
τ_{00}	0.08 $_{BatID}$			0.04 $_{BatID}$			0.00 $_{BatID}$		
ICC	0.20			0.19			0.13		
n	28 $_{BatID}$			28 $_{BatID}$			21 $_{BatID}$		
Observations	684			648			438		
R^2m / R^2c	0.16 / 0.33			0.39 / 0.51			0.86 / 0.88		

Table S2 Generalized linear mixed-effect models with Gamma family and log link. Reproductive status and state (torpid or resting) explained variation in $\dot{V}O_2$, f_H and T_{skin}. Individual (BatID) was included as a random effect. Reported p-values are based on t-statistics and use the normal distribution function (Lüdecke 2021)

	$\dot{V}O_2$			f_H			T_{skin}		
**Predictors**	Estimates	CI	p	Estimates	CI	p	Estimates	CI	p
Non-Reproductive Torpid	0.11	0.08 – 0.16	**<0.001**	19.31	16.22 – 22.98	**<0.001**	10.87	10.11 – 11.69	**<0.001**
Reproductive Torpid	4.15	2.36 – 7.28	**<0.001**	3.68	2.77 – 4.90	**<0.001**	1.65	1.46 – 1.86	**<0.001**
Reproductive Resting	38.13	22.36 – 65.00	**<0.001**	17.74	13.49 – 23.33	**<0.001**	2.37	2.11 – 2.66	**<0.001**
**Random Effect [BatID]**									
σ^2	0.29			0.08			0.02		
τ_{00}	0.16 BatID			0.03 BatID			0.00 BatID		
ICC	0.35			0.28			0.20		
N	31 BatID			31 BatID			31 BatID		
Observations	715			715			715		
R^2m / R^2c	0.839 / 0.896			0.927 / 0.948			0.866 / 0.893		

Table S3 The linear mixed-effect model that included f_H has a higher predictive ability for $\dot{V}O_2$ compared to the model including T_{skin}

Model	AICc	R^2m	R^2c
$\log(\dot{V}O_2) \sim f_H + (1\| BatID)$	4510.0	0.65	0.83
$\log(\dot{V}O_2) \sim T_{skin} + (1\|BatID)$	6222.5	0.31	0.56

Figure S1 Representative figures of two bats during the 6 h-experiment. **a)** Upper panel: The bat remains torpid and $\dot{V}O_2$ (black dashed line) and f_H (pink solid line) are low across the temperature range. Lower panel: The bat thermoconforms T_{skin} (light blue solid line) to T_a (dark blue dashed line). **b)** Upper panel: The bat enters torpor and briefly arouses around 10:00 and $\dot{V}O_2$ (black dashed line) and f_H (pink solid line) increase until the bat enters torpor again. Lower panel: The bat thermoconforms T_{skin} (light blue solid line) to T_a (dark blue dashed line) and the brief arousal is not reflected in a distinct increase of T_{skin} (Online version in colour.)

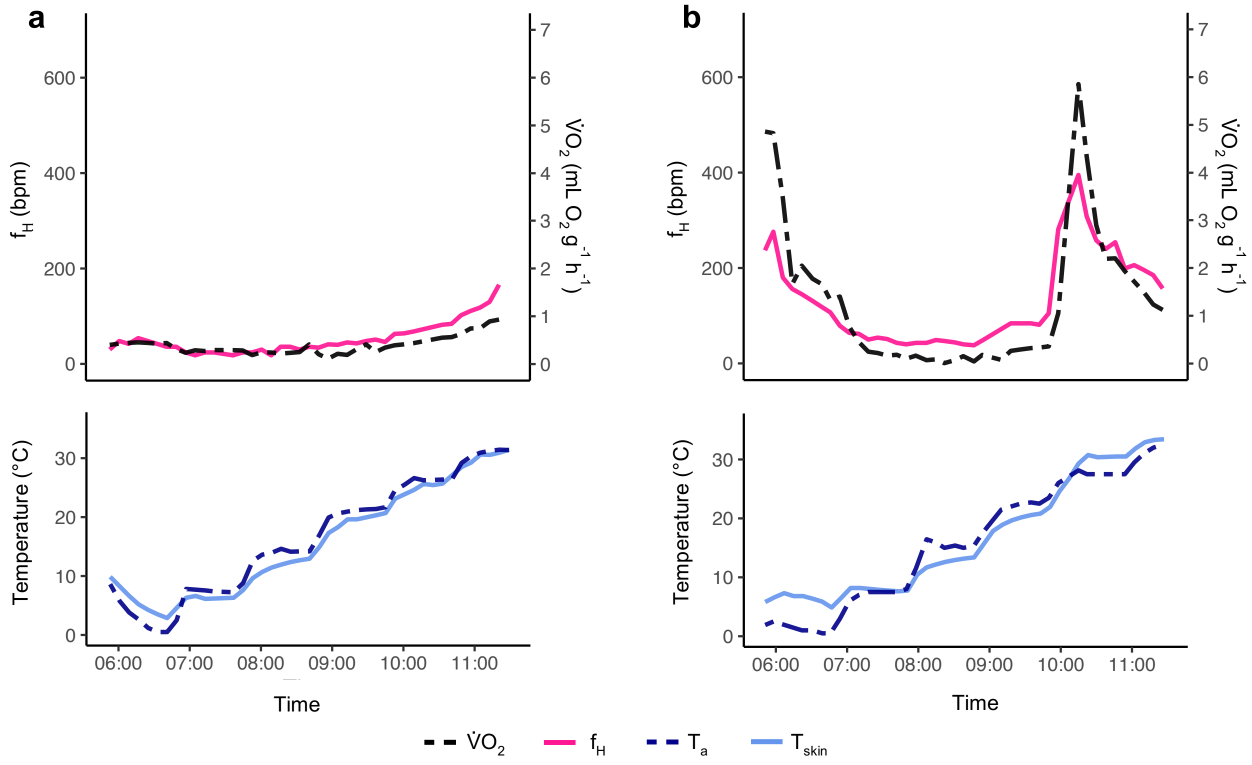

Figure S2 Representative figure of a reproductive bat using the "Combination" torpor use strategy in the 12 h-experiment**.** Upper panel: $\dot{V}O_2$ (black dashed line) and f_H (pink solid line) are lowered after a 2 h resting period and the bat is torpid until arousal around 13:00. The bat remains resting until the end of the experiment. Lower panel: When torpid, the bat thermoconforms T_{skin} (light blue solid line) to T_a (dark blue dashed line) and when resting T_{skin} is higher compared to T_a (Online version in colour.)

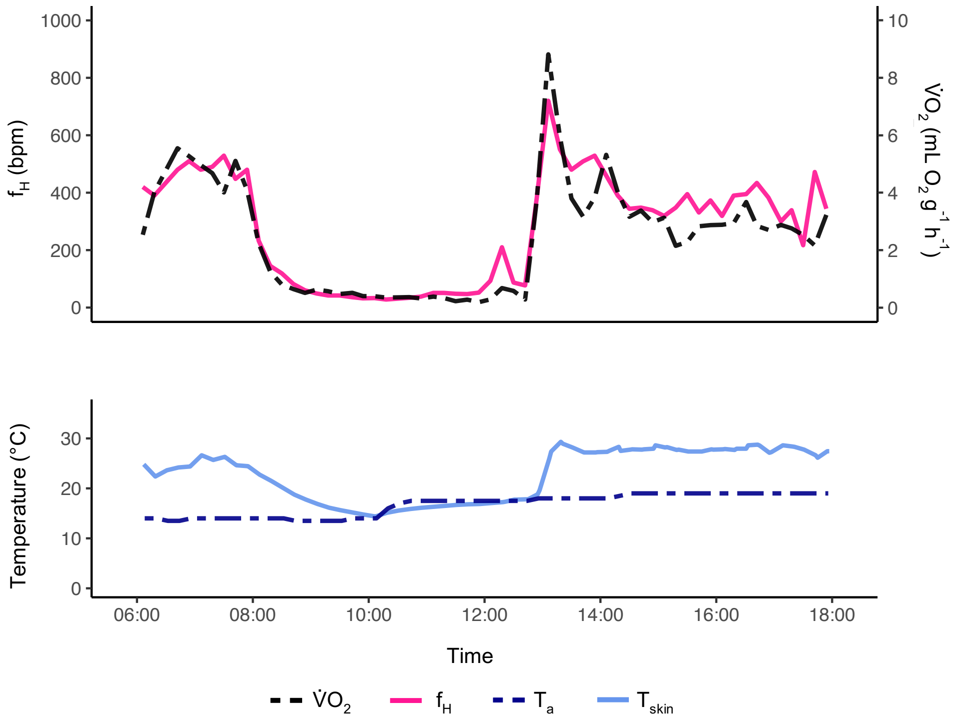

References

Lüdecke D (2021) sjPlot: Data Visualization for Statistics in Social Science. https://CRAN.R-project.org/package=sjPlot
